# Single-Cell Transcriptomic Analysis Reveals Copper Metabolism Heterogeneity and Phenotypic Remodeling in Vaginal Anterior Wall Stromal Cells in Pelvic Organ Prolapse

**DOI:** 10.3390/biology15201841

**Published:** 2026-10-14

**Authors:** Zhi Wang, Shouqi Huang, Ming Hu, Hongyang Xue, Jianming Tang, Jie Min, Li Hong, Suting Li

**Affiliations:** Department of Gynecology and Obstetrics, Renmin Hospital of Wuhan University, Wuhan 430060, China; wangzhi6666@whu.edu.cn (Z.W.); 2020305231018@whu.edu.cn (S.H.); rena@whu.edu.cn (M.H.); 2018305230034@whu.edu.cn (H.X.); tangjianming2009@whu.edu.cn (J.T.); jie_min@whu.edu.cn (J.M.)

**Keywords:** pelvic organ prolapse, single-cell transcriptome, copper metabolism, extracellular matrix, fibroblast heterogeneity, cellular senescence

## Abstract

Pelvic organ prolapse (POP) is a common disease characterized by disordered extracellular matrix (ECM) remodeling of pelvic support tissues. Copper dysregulation drives fibrosis in multiple diseases, whereas its function in POP remains unclear. We analyzed single-cell transcriptomes of 90,578 cells from 21 vaginal wall samples (5 controls, 16 POP). ECM remodeling was the most robustly upregulated module in POP fibroblasts, accompanied by decreased copper metabolism and senescence scores. Fibroblasts resolved into nine subpopulations, with copper metabolism activity decreased in eight, most prominently in p53/senescence-like states. High-copper fibroblasts were enriched for TNFα/NF-κB, inflammatory, hypoxic, and EMT programs. Copper metabolism correlated with senescence (rho = 0.56, *p* = 0.010) but not ECM remodeling (rho = −0.02, *p* = 0.92). Smooth muscle cells shifted toward a synthetic phenotype, negatively correlating with copper metabolism. CellChat and trajectory inference revealed ECM ligand-driven interactions and temporal copper–ECM gene dynamics. Elesclomol reduced fibroblast viability and induced apoptosis and senescence in a copper-dependent manner. These findings suggest that copper metabolism dysregulation contributes to POP stromal remodeling and may serve as a potential therapeutic target.

## Simple Summary

Pelvic organ prolapse (POP) is a common disease among middle-aged and elderly women. The weakening of pelvic support tissues serves as the main cause, but the mechanisms remain incompletely understood. Copper is essential for humans. In this study, we analyzed the transcriptional profiles of 90,578 cells from the vaginal wall tissues of 21 samples (5 controls and 16 POP) using single-cell RNA sequencing data. We found that extracellular matrix (ECM) remodeling was significantly enhanced in POP, while copper metabolism was decreased in fibroblasts. Copper metabolism was positively correlated with senescence but not with ECM remodeling programs. Fibroblasts were resolved into nine subpopulations, eight of which exhibited decreased copper metabolism score, with the greatest decrease observed in the p53/senescence-like state. High-copper fibroblasts were enriched for inflammatory and hypoxic programs. Smooth muscle cells shifted toward a synthetic phenotype, and their copper metabolism activity was negatively correlated with synthetic scores. In vitro experiments showed that the copper ionophore Elesclomol reduced fibroblast viability and induced apoptosis and senescence in a copper-dependent manner. Our findings suggest that abnormal copper metabolism may be involved in the pathogenesis of POP. This insight may help inform future strategies for the prevention and treatment of POP.

## 1. Introduction

Pelvic Organ Prolapse (POP) is a type of female pelvic floor dysfunction characterized by the descent of pelvic viscera into or beyond the vagina. As a major chronic disease, POP imposes a substantial burden on women’s physical and mental health and on healthcare systems worldwide [1,2]. According to the Global Burden of Disease data spanning 1990 to 2021, the age-standardized incidence, mortality and disability-adjusted life year (DALY) rates of POP worldwide have gradually declined, whereas the absolute numbers of cases, deaths and DALY burdens keep rising [3]. Middle-aged and elderly women constitute the high-risk population for POP. Although the age-standardized risk has declined, global population aging is continuously expanding the absolute number of affected individuals. It is projected that 156 million women worldwide will suffer from POP by 2036, making POP a persistent global public health challenge [4]. At present, surgery remains the primary treatment for moderate and severe POP. Despite advances in surgical techniques, the postoperative recurrence rate stays relatively high [5]. This indicates that our understanding of POP pathogenesis remains incomplete, and effective targeted intervention strategies are still lacking at this stage.

The integrity of pelvic supportive structures relies on the dynamic balance of extracellular matrix (ECM) within connective tissues including the vaginal wall, cardinal ligaments and uterosacral ligaments. The ECM is mainly composed of collagens, elastin and proteoglycans, which endow pelvic floor tissues with tensile strength and elasticity. Fibroblasts serve as the primary producers of ECM, and their functional disturbance constitutes a core pathological event of POP [6]. Smooth muscle cells (SMCs) modulate pelvic tissue tension via contraction and relaxation. During POP progression, SMCs may undergo phenotypic switching from a contractile phenotype to a synthetic/fibrotic phenotype, which further drives aberrant ECM remodeling [7,8,9].

Copper is an essential element for humans. As a cofactor of multiple enzymes including lysyl oxidase (LOX), superoxide dismutase 1 and cytochrome c oxidase, copper participates in redox reactions, mitochondrial respiration and ECM crosslinking [10,11,12]. In recent years, the role of disrupted copper metabolism in fibrotic diseases has attracted growing research attention. Copper activates LOX to facilitate crosslinking of collagen and elastin, thereby triggering ECM stiffening and fibrosis [12,13]. Conversely, excessive copper accumulation induces oxidative stress and mitochondrial dysfunction, which further initiate cellular senescence and inflammatory responses [11,13]. Nevertheless, systematic single-cell resolution analysis of copper metabolic heterogeneity in POP is still lacking.

Single-cell RNA sequencing is a powerful tool for dissecting intratissue cellular heterogeneity and disease pathological mechanisms. GSE151192 is a single-cell transcriptomic dataset obtained from vaginal wall tissues of patients with POP [14]. In this study, we analyzed single-cell transcriptomes of 90,578 cells from 21 vaginal wall samples to systematically characterize copper metabolic heterogeneity in vaginal stromal cells and its association with ECM remodeling, cellular senescence, SMCs phenotypic switching, and fibroblast subpopulation reprogramming. We further validated the functional impact of copper homeostasis imbalance on fibroblast viability, apoptosis, and senescence using Elesclomol (ES) and copper co-treatment in L929 fibroblasts. This study provides novel insights into the molecular pathogenesis of POP and identifies promising therapeutic targets for clinical application.

## 2. Materials and Methods

### 2.1. Data Source and Preprocessing

Human vaginal wall single-cell transcriptome data were retrieved from the Gene Expression Omnibus (GEO) database under accession number GSE151192, comprising 21 samples (5 controls and 16 POP; 90,578 cells after quality control) [14]. Raw sequencing reads were aligned and quantified using Cell Ranger (v3.0.2) with the hg38 human reference genome. All downstream analyses were performed in R with the Seurat package (v5.0.0). Cells that satisfied the following quality control criteria were retained: the number of detected RNA features (nFeature_RNA) ranged from 200 to 5000, and the percentage of mitochondrial gene counts was less than 20%. Gene expression normalization was implemented via the LogNormalize function with a scale factor of 10,000. The top 2000 highly variable genes were identified, followed by ScaleData transformation, in which the percentage of mitochondrial reads (percent.mt) and total RNA counts per cell (nCount_RNA) were regressed out. Batch effects across datasets and individual samples were corrected by the Harmony algorithm. Uniform Manifold Approximation and Projection (UMAP) dimensionality reduction and clustering (resolution = 0.6) were conducted to annotate distinct cell populations.

### 2.2. Cell Type Annotation

Cell type annotation was performed by calculating the average z-score expression of canonical marker genes within each cluster and assigning each cluster to the cell type with the highest score. Marker genes were derived from CellMarker 2.0 [15], PanglaoDB [16], and original tissue-specific literature, as detailed below:

Fibroblast: *COL1A1*, *COL1A2*, *DCN*, *FAP*;

Smooth muscle cell: *ACTA2*, *TAGLN*, *MYH11*;

Macrophage: *CD68*, *CD163*, *CSF1R*;

T cell: *CD3D*, *CD3E*, *CD2*;

B cell: *MS4A1*, *CD79A*;

Endothelial cell: *PECAM1*, *VWF*, *CDH5*;

Epithelial cell: *EPCAM*, *KRT5, KRT14*;

Adipocyte: *LPL*, *ADIPOQ*;

Mast cell: *TPSAB1*, *MS4A2*;

Neuronal cell: *NRXN1*, *PCSK2*;

Proliferating cell: *DLGAP5*, *RRM2*.

### 2.3. Custom Gene Sets and Module Score Calculation

Gene sets related to copper metabolism, ECM remodeling, cellular senescence, mitochondrial damage, and cuproptosis were compiled from published literature (Table 1). The complete member lists of the five gene sets, functional annotations of each gene, and their presence in the reference gene sets used are provided in Supplementary Tables S1–S6. To reduce dependence on any single scoring algorithm, each module was evaluated using four independent methods:

- AddModuleScore (Seurat)—average expression of the gene set minus matched control features.
- AUCell—rank-based scoring, robust to sequencing depth.
- Pseudobulk z-score—module scores aggregated to 21 samples; between-group comparisons performed using the Wilcoxon test (n = 5 vs. 16).
- Depth-matched scoring—module scores calculated within nFeature-stratified bins and standardized within bins to control for the correlation between sequencing depth and scores.

A module was considered robust only when the direction of the POP versus control difference was consistent across at least three of the four methods.

### 2.4. Fibroblast Subpopulation Analysis

Fibroblasts were extracted from the dataset and re-clustered using the Harmony-corrected dimensionality reduction embedding (top 30 dimensions) at a resolution of 0.6, yielding nine fibroblast subpopulations (F1–F9). Subpopulations were annotated based on FindAllMarkers output (Wilcoxon rank-sum test, only.pos = TRUE, min.pct = 0.25, logfc.threshold = 0.5) and manually curated against canonical marker genes.

For each subpopulation, copper metabolism module scores were compared between POP and control at the patient level (pseudobulk mean per subject; Wilcoxon rank-sum test). Two additional copper-related scores calculated on the same cells—AddModuleScore-based (AMS_Copper_1) and AUCell-based (AUC_Copper)—served as robustness checks, and a cuproptosis score (Cuproptosis_Score) was simultaneously calculated to test whether the copper metabolism module and the cuproptosis module behaved consistently.

To identify transcriptional programs associated with copper metabolic activity within fibroblasts, cells were stratified using a within-patient median split of the copper metabolism score: for each of the 21 subjects, fibroblasts above that subject’s median were labeled “high” and those below were labeled “low,” thereby ensuring that both groups were derived from the same patients and that comparisons were not confounded by inter-patient variation. Differential expression between high- and low-copper fibroblasts was calculated using FindMarkers and submitted to fgsea for comparison against MSigDB Hallmark and GO Biological Process collections.

### 2.5. Phenotypic Analysis of SMCs

A single fixed gene set was used consistently across all analyses, tables, and figures to establish two signature scores: a contractile score (*ACTA2*, *TAGLN*, *MYH11*, *CNN1*, *MYLK*, *LMOD1*, *TPM2*, *CALD1*) and a synthetic score (*VCAN*, *COL1A1*, *COL3A1*, *FN1*, *MMP2*, *SPP1*, *POSTN*, *TGFB1*, *CTGF*, *PALLD*). The phenotypic index was defined as the synthetic score minus the contractile score.

### 2.6. Cell–Cell Communication Analysis

Ligand-receptor interactions between cell populations were inferred using CellChat (version 1.6.0). Cell communication networks were separately constructed for control and POP groups, followed by comparisons of overall communication strength and signaling pathway activity. To explore subpopulation-specific signaling among fibroblasts, each fibroblast subcluster was treated as an independent cell type for a secondary CellChat analysis.

### 2.7. Construction of Ligand–Target Regulatory Network

Ligand–target regulatory networks were built based on top-ranked ligands from CellChat and upregulated differentially expressed genes (DEGs) in fibroblasts. Regulatory interactions were curated from published fibrotic signaling pathway literature.

### 2.8. Trajectory Inference and Dynamic Gene Analysis

Slingshot algorithm was applied to perform pseudotime trajectory analysis on fibroblasts. TradeSeq was used to identify genes with significantly variable expression along pseudotime, with special focus on expression dynamics of copper metabolism and ECM-related genes.

### 2.9. Cross-Module Correlation

Correlation analyses of copper metabolism, ECM remodeling, senescence, and mitochondrial scores were performed using the AUCell implementation, ensuring that all metrics were generated by the same algorithm and were therefore directly comparable. Correlations were calculated at two levels: (1) the cell level, for descriptive purposes only; and (2) the patient level, where module scores were first averaged within each subject (pseudobulk) and Spearman’s rho was then calculated across the 21 subjects, and repeated separately within the POP and control strata.

### 2.10. Cellular Experiments

The L929 mouse fibroblast cell line used in this study was obtained from Procell Biotechnology (Cell No. PCH 2025061110, Wuhan, China). The cells were authenticated by short tandem repeat (STR) profiling prior to experimental use, and the obtained STR profile matched the reference profile of NCTC clone 929 (L929) in the Cellosaurus database with a match rate of 96.1%. The cells were tested negative for mycoplasma contamination. No commonly misidentified cell lines were used in this study. All cell experiments were independently repeated three times.

#### 2.10.1. CCK-8 Cell Viability Assay

Cell viability and proliferative capacity of L929 fibroblasts were assessed using the CCK-8 assay. Experiments were divided into two series: a copper-free series consisting of a control group and ES (HY-12040, MCE, Monmouth Junction, NJ, USA) at 25, 50, 100, and 200 nM (5 groups), and a copper-supplemented series with the same ES concentrations plus 15 µM CuCl_2_ (HY-107972, MCE, Monmouth Junction, NJ, USA), yielding 10 groups in total, with 3 replicate wells per group. Cells from each group were evenly seeded into 96-well culture plates and continuously treated for 24 h. The original culture medium was discarded, and fresh medium supplemented with CCK-8 reagent (40203ES80, Yeasen Biotechnology (Shanghai) Co., Ltd., Shanghai, China) was added to each well. Plates were incubated in a constant-temperature incubator away from light for 2 h. Absorbance (OD value) at 450 nm was measured using a PerkinElmer Victor3 1420 Multilabel Counter (PerkinElmer, Waltham, MA, USA), and changes in cell viability were evaluated based on OD readings.

#### 2.10.2. Intracellular Copper Quantification

Intracellular copper ion content was measured using a copper colorimetric assay kit (E-BC-K300-M, Elabscience Biotechnology Co., Ltd., Wuhan, China). Experiments were divided into 8 groups: control, CuCl_2_ alone (15 µM), ES 25 nM, ES 50 nM, ES 100 nM, Cu + ES 25 nM, Cu + ES 50 nM, and Cu + ES 100 nM, with 3 replicate wells per group. Cells were digested with EDTA-free trypsin, lysed, and the supernatant was collected for subsequent detection. Before detection, reagents were equilibrated to room temperature and Reagent 1 was incubated at 37 °C until clear. Reagent 2 working solution was prepared immediately before use, and the chromogenic working solution was prepared by mixing Reagent 1 and Reagent 2 working solution at a 14:1 volume ratio. A 100 μmol/L copper standard was serially diluted with double-distilled water. Subsequently, 20 μL of standard or sample was added to each well of a 96-well plate, followed by 200 μL of chromogenic working solution. After shaking for 20 s and covering with a membrane, the plate was incubated at 37 °C for 30 min. Absorbance was measured at 580 nm using a PerkinElmer Victor3 1420 Multilabel Counter (PerkinElmer, Waltham, MA, USA), and copper ion concentrations were calculated from the standard curve.

#### 2.10.3. Apoptosis Detection via Flow Cytometry

Apoptotic rates of L929 fibroblasts were quantified by Annexin V-FITC/PI double-staining flow cytometry. After treatment with CuCl_2_ 15 µM combined with ES at 25, 50, or 100 nM for 24 h, cells were harvested, digested with trypsin, pelleted by centrifugation, and thoroughly washed with pre-cooled PBS. According to the apoptosis detection kit instructions, Annexin V-FITC and PI staining solutions (40302ES50, Yeasen Biotechnology (Shanghai) Co., Ltd., Shanghai, China) were sequentially added for light-shielded staining. Stained samples were analyzed by flow cytometry, and the proportion of apoptotic cells was quantified and compared across groups to clarify the impact of copper overload on fibroblast apoptosis.

#### 2.10.4. Senescence-Associated β-Galactosidase (SA-β-Gal) Staining

SA-β-Gal staining was performed to observe the senescent phenotype of L929 fibroblasts. After treatment with CuCl_2_ 15 µM combined with ES 50 nM for 24 h, culture medium was removed, cells were rinsed with PBS and fixed with fixative solution at room temperature. The fixative was aspirated, cells were washed again, and freshly prepared SA-β-Gal working staining solution (G1073-100T, Servicebio, Wuhan, China) was added. Samples were incubated overnight at 37 °C under CO_2_-free and light-shielded conditions. The next day, cellular staining was visualized under a microscope (BX53, Olympus Corporation, Tokyo, Japan); blue-stained cells were defined as senescence-positive cells. Multiple random fields were captured to calculate the percentage of senescent cells and assess copper overload-induced cellular senescence.

### 2.11. Statistical Analysis

Wilcoxon rank-sum test was adopted for comparisons of continuous variables. Spearman’s correlation analysis was used to evaluate pairwise correlations. The BH method was applied to correct *p*-values for multiple comparisons. All bioinformatic analyses were implemented in R v4.6.0. Measurement data were expressed as mean ± standard deviation (mean ± SD). Independent sample *t*-test was used for two-group comparisons; one-way analysis of variance (ANOVA) was performed for overall differences among multiple groups, followed by Tukey’s post hoc test for pairwise intergroup comparisons. GraphPad Prism 11 software was used for all statistical calculations. Statistical significance threshold was set at *p* < 0.05. All cellular functional experiments were independently repeated three times.

## 3. Results

### 3.1. Single-Cell Atlas of Vaginal Wall Tissues

**Figure 1:**
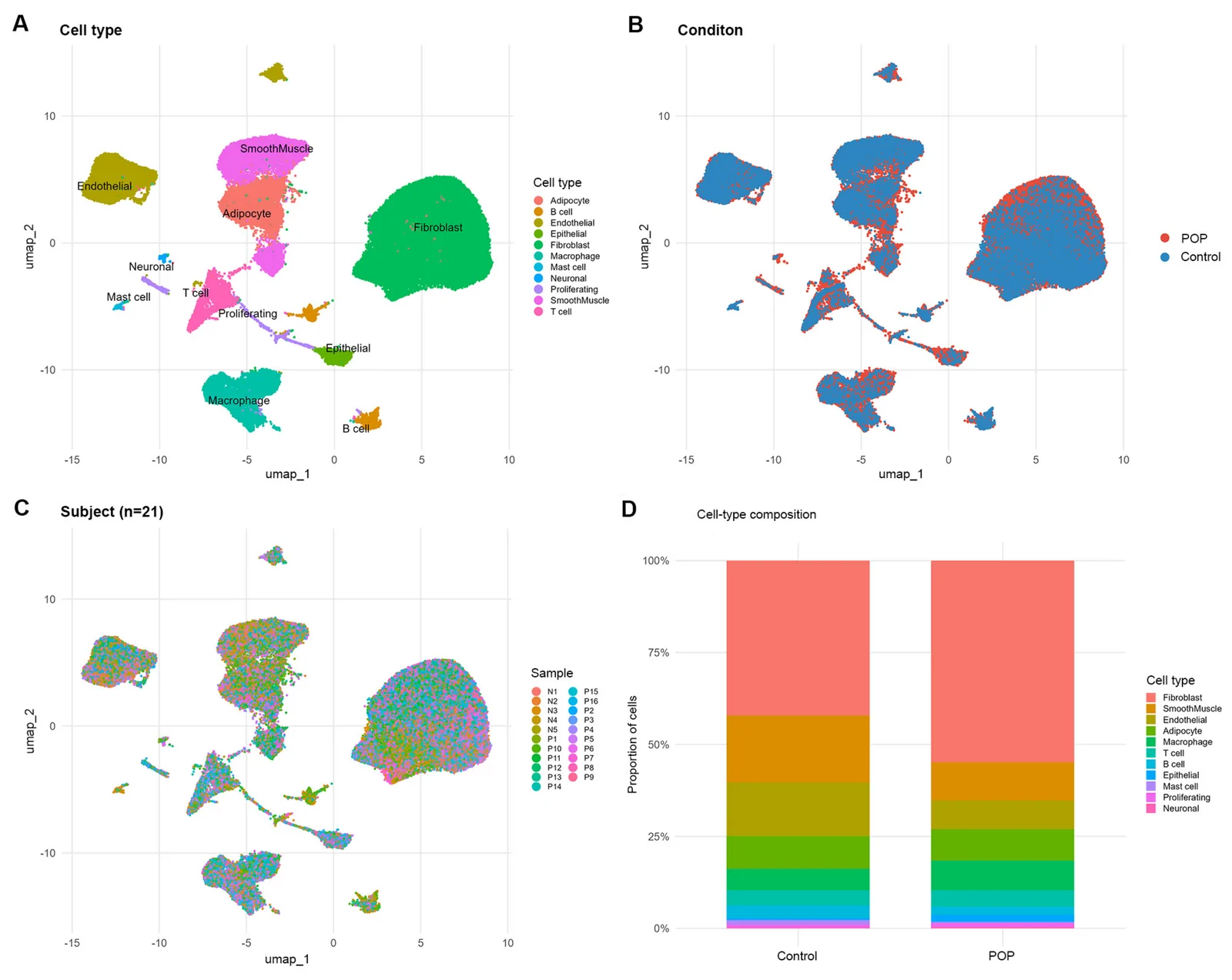
Single-cell atlas of the human vaginal wall. (**A**) UMAP embedding of 90,578 cells (21 samples: 5 controls, 16 POP), colored by annotated cell type (11 populations). (**B**) The same embedding colored by group (control vs. POP). (**C**) The same embedding colored by subject, showing that cell type structure is not driven by any single donor. (**D**) Cell type composition of the control and POP groups (proportion of all cells).

After quality control, 90,578 high-quality cells from 21 samples (5 controls and 16 POP) were retained. UMAP dimensionality reduction resolved 11 distinct cell populations, annotated based on canonical marker gene expression (Figure 1A–D). Fibroblasts were the predominant population, followed by SMCs, endothelial cells, macrophages, and adipocytes; T cells, B cells, epithelial cells, and other minor types accounted for smaller proportions.

Table 2 shows the proportions of cell types among different groups. The proportion of fibroblasts was higher in the POP group than in controls, while SMCs decreased and that of macrophages increased. These shifts in cell composition suggest that dynamic remodeling of stromal and immune cells is an important feature of POP. However, none of the differences reached statistical significance, indicating a trend toward stromal remodeling in POP that requires confirmation in a larger cohort.

### 3.2. Fibroblast Module Scores and Subpopulation Heterogeneity

Fibroblasts are the primary producers of ECM. We therefore focused on evaluating five functional modules—copper metabolism, ECM remodeling, cellular senescence, mitochondrial damage, and cuproptosis—using four independent scoring methods (Section 2.3; Table 3).

The results showed that ECM remodeling was the only module robustly elevated in POP fibroblasts across all four methods (AddModuleScore *p* = 1.7 × 10^−168^; AUCell *p* = 4.4 × 10^−253^; pseudobulk and depth-matched scores were also elevated), consistent with the well-established role of ECM remodeling in POP pathogenesis. Copper metabolism scores were robustly decreased in POP fibroblasts, being lower by AddModuleScore (*p* ≈ 0), AUCell (*p* ≈ 0), and depth-matched scoring (*p* = 1 × 10^−5^), with only the pseudobulk z-score failing to reach significance (*p* = 0.40). Cellular senescence was also lower in POP fibroblasts across all four methods (AddModuleScore *p* ≈ 0; AUCell *p* ≈ 0; depth-matched *p* = 1 × 10^−5^; pseudobulk *p* = 0.35). Mitochondrial damage and cuproptosis showed inconsistent directions across methods and were therefore not interpreted.

### 3.3. Copper Metabolism Activity Is Broadly Decreased Across Fibroblast Subpopulations

**Figure 2:**
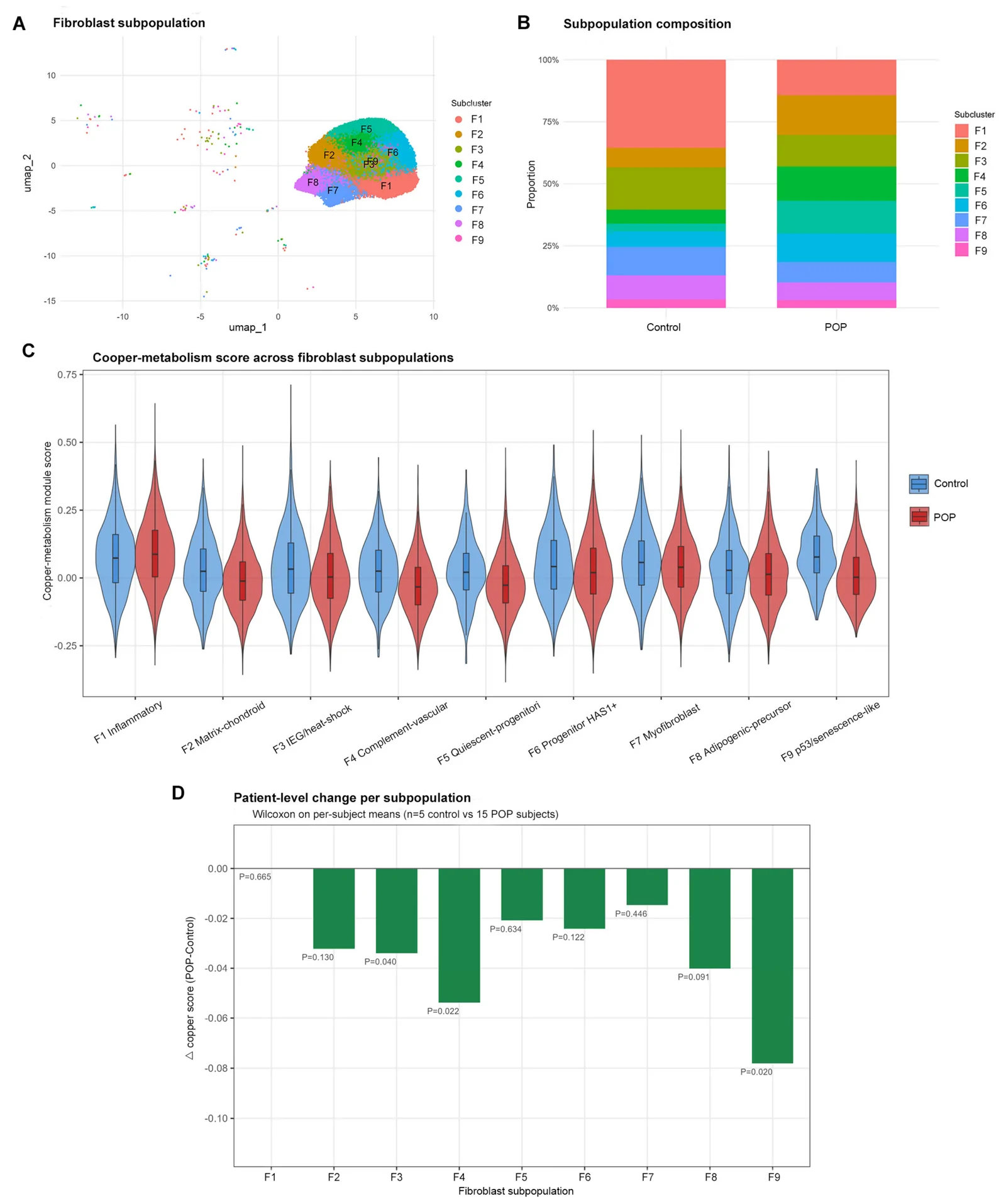
Module scoring and subpopulation structure of fibroblasts. (**A**) UMAP of the fibroblast compartment showing nine subpopulations (F1–F9). (**B**) Relative abundance of each subpopulation in control and POP. (**C**) Cell-level copper metabolism scores for each of the nine fibroblast subpopulations (F1–F9). (**D**) Patient-level changes in copper metabolism scores for each subpopulation (POP—control; Wilcoxon test on per-subject means, n = 5 controls vs. 16 POP) with *p* values.

As shown in Table 4, re-clustering of the fibroblast compartment resolved nine subpopulations (F1–F9), whose functional identities were determined based on marker genes (Section 2.4; Figure 2A,B; full annotations in Supplementary Table S7).

The composition of the fibroblast compartment underwent substantial remodeling in POP, although only one state reached nominal significance at the patient level (Supplementary Tables S8 and S9). The inflammatory/stress state F1 contracted markedly (median proportion of subject-derived fibroblasts: control 25.2% vs. POP 10.6%; Δ = −14.6 percentage points; *p* = 0.091, FDR = 0.164), whereas the matrix/chondroid-like state F2 expanded (control 7.5% vs. POP 16.0%; Δ = +8.5 percentage points; *p* = 0.015, FDR = 0.066), and the immediate early gene state F3 contracted (control 17.2% vs. POP 12.3%; *p* = 0.006, FDR = 0.053). The progenitor states F5 and F6 also expanded (+7.5 and +5.4 percentage points; *p* = 0.080 and *p* = 0.062, respectively). Although these changes were directionally clear and considerable in magnitude, most individual comparisons did not pass FDR correction; we therefore describe this change as a trend toward replacement of inflammatory and immediate early gene states by matrix-producing and progenitor states, rather than as an established set of compositional differences. We explicitly note that, within the same dataset, testing subpopulation abundance at the cell level (chi-square test across all 47,437 fibroblasts) drove all nine states to *p* ≈ 0; this illustrates the pseudoreplication that patient-level testing avoids, and is precisely why patient-level values were used throughout this study.

We next scored copper metabolism program separately in the nine states (patient-level pseudobulk; Figure 2C,D; Supplementary Table S10). This score was lower in POP in 8 of the 9 states, with the only exception being the inflammatory state F1, which remained essentially unchanged (Δ = +0.0001, *p* = 0.66). The largest decreases were observed in the p53/senescence-like state F9 (Δ = −0.078, *p* = 0.020) and the complement/vascular state F4 (Δ = −0.054, *p* = 0.022), followed by the adipogenic precursor state F8 (Δ = −0.040, *p* = 0.091), the immediate early gene state F3 (Δ = −0.034, *p* = 0.040), and the matrix/chondroid-like state F2 (Δ = −0.032, *p* = 0.130). When implemented with AddModuleScore, F4 and F9 remained nominally significant (*p* = 0.016 and *p* = 0.020, respectively) with trends preserved; when implemented with rank-based AUCell, only F9 remained nominally significant (*p* = 0.020), while trends in the remaining states were attenuated. The cuproptosis score showed the opposite direction (F9 +0.016, *p* = 0.18), confirming that the copper metabolism module and the cuproptosis module are not interchangeable, and that the present results concern copper handling rather than copper-dependent cell death.

### 3.4. High-Copper Fibroblast States Are Defined by Inflammatory Programs Rather than Matrix Programs

**Figure 3:**
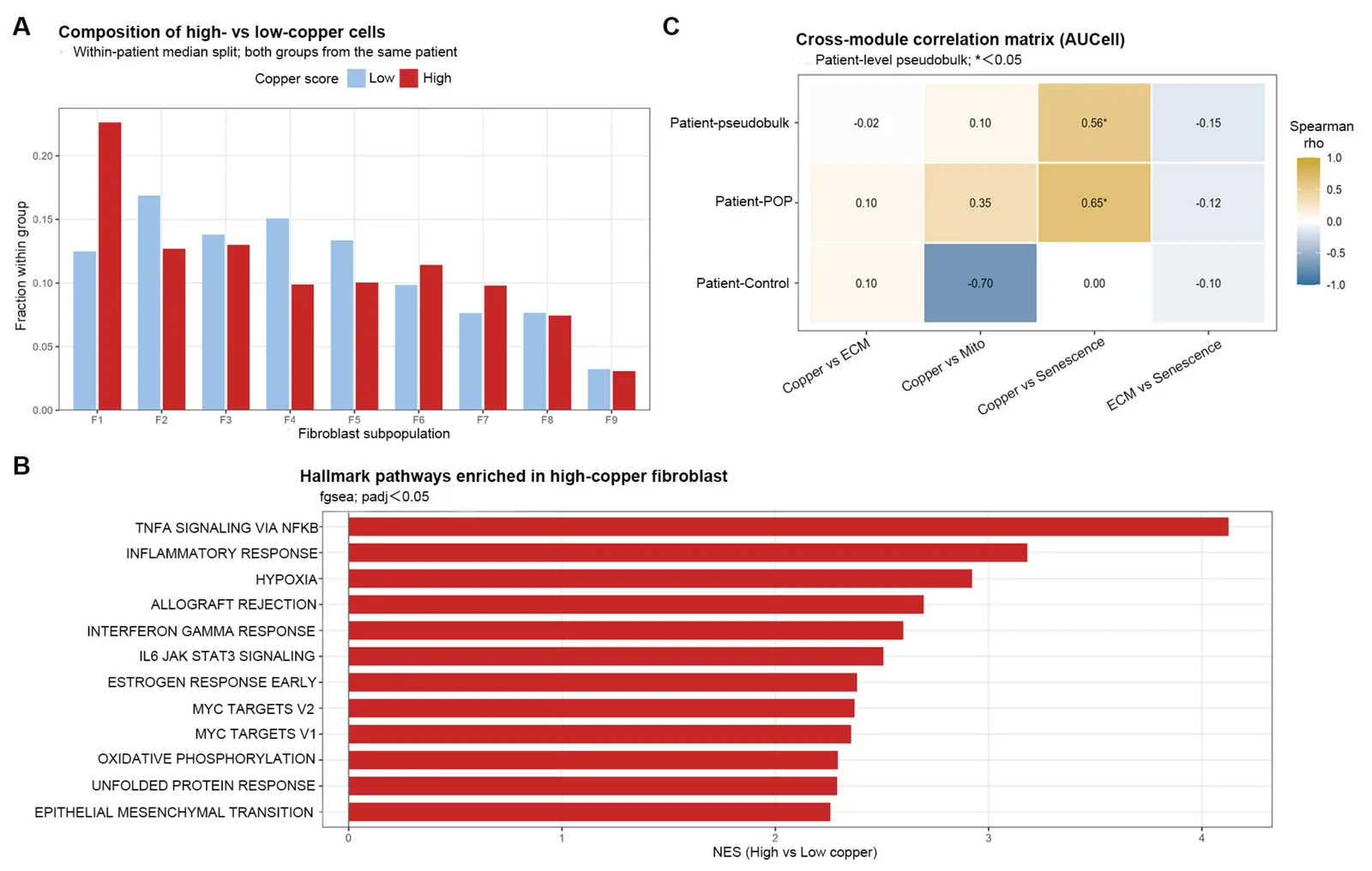
High-copper fibroblast states are defined by inflammatory programs rather than matrix programs. (**A**) Composition of high- vs. low-copper cells across fibroblast subpopulations. Within each patient, fibroblasts were divided into high-copper (red) and low-copper (blue) groups using a within-patient median split of the copper metabolism score; the proportion of cells from each group across subpopulations (F1–F9) is shown as bar plots. (**B**) Hallmark pathway enrichment analysis of high-copper fibroblasts. Pathway enrichment of DEGs was performed using fgsea, showing representative pathways with padj < 0.05. (**C**) Cross-module correlation matrix of copper metabolism with ECM remodeling, mitochondrial, and senescence module scores (AUCell implementation, patient-level pseudobulk). Colors represent Spearman’s rho, with yellow indicating positive correlation and blue indicating negative correlation. The matrix is displayed separately for all patient-level samples (patient-pseudobulk, n = 21), within the POP stratum (patient-POP, n = 16), and within the control stratum (patient-Control, n = 5). * *p* < 0.05.

To characterize what distinguishes fibroblasts with high copper metabolism activity, we stratified cells using a within-patient median split of the copper metabolism score (23,724 “high” and 23,713 “low” cells; each of the 21 subjects contributed approximately equal numbers of cells to both groups). This design eliminates inter-patient variation by construction, so any differences reflect cell state rather than patient identity. Figure 3A shows the proportion of cells from the two groups across the nine subpopulations. High-copper cells were markedly enriched in the inflammatory/stress state F1, suggesting a coupling between copper metabolism activity and inflammatory programs at the level of cell state. Conversely, the p53/senescence-like state F9 had the lowest proportion of high-copper cells, indicating that cells with the highest copper metabolism activity are not in a senescent-like state. Differences in the proportions of high- and low-copper cells in the remaining subpopulations were relatively small, indicating that copper metabolism is not uniformly distributed across all fibroblast states but is associated with specific functional states.

Differential expression yielded 3213 genes (805 upregulated and 387 downregulated in high-copper fibroblasts; Supplementary Table S11). Comparison of fgsea results against the Hallmark collection returned 26 of 50 pathways with padj < 0.05, almost all positively enriched (Figure 3B; Supplementary Table S12). The single dominant signal was TNFA_SIGNALING_VIA_NFKB (NES = 4.12, padj = 1.0 × 10^−31^), followed by INFLAMMATORY_RESPONSE (NES = 3.18), HYPOXIA (2.92), ALLOGRAFT_REJECTION (2.70), INTERFERON_GAMMA_RESPONSE (2.60), and IL6_JAK_STAT3_SIGNALING (2.51). Epithelial–mesenchymal transition (NES = 2.26), OXIDATIVE_PHOSPHORYLATION (2.29), MTORC1_SIGNALING (2.10), and TGF_BETA_SIGNALING (1.82) were also enriched. Comparison against the GO Biological Process collection (Supplementary Table S13) identified 275 significant sets out of 1,911, led by INORGANIC_ION_HOMEOSTASIS (NES = 3.19), DETOXIFICATION (3.09), MONOATOMIC_ION_HOMEOSTASIS (3.04), RESPONSE_TO_CADMIUM_ION (3.03), and INTRACELLULAR_ZINC_ION_HOMEOSTASIS (2.97).

Together, these analyses indicate that high copper metabolism activity in fibroblasts is coupled to inflammatory and hypoxic signaling rather than to matrix production—consistent with the absence of a copper-ECM correlation and with the observation that the inflammatory state F1 is the only state in which the copper metabolism score did not decrease.

### 3.5. Copper Metabolism in Fibroblast States Covaries with Senescence, Not with ECM Modules

To place the copper metabolism in the context of other modules, we correlated it with ECM remodeling, senescence, and mitochondrial damage, all generated using the AUCell implementation to ensure direct comparability across metrics (Figure 3C; Supplementary Table S14). At the patient level (n = 21), the copper metabolism score was positively correlated with the senescence score (rho = 0.56, *p* = 0.010), whereas it showed no relationship with the ECM remodeling score (rho = −0.02, *p* = 0.92) or the mitochondrial score (rho = 0.10, *p* = 0.67). The copper–senescence association was stronger within the POP (n = 16, rho = 0.65, *p* = 0.008) and absent within the control (n = 5, rho = 0.00). Copper metabolic transcriptional activity in POP fibroblasts thus covaries with senescence programs rather than matrix programs, consistent with the senescence-like state F9 showing the largest decrease in copper score and with high-copper cells being enriched for inflammatory and hypoxic rather than collagen programs. This also explains why ECM-high subpopulations do not carry a copper co-signature.

### 3.6. SMCs Phenotypic Reprogramming

**Figure 4:**
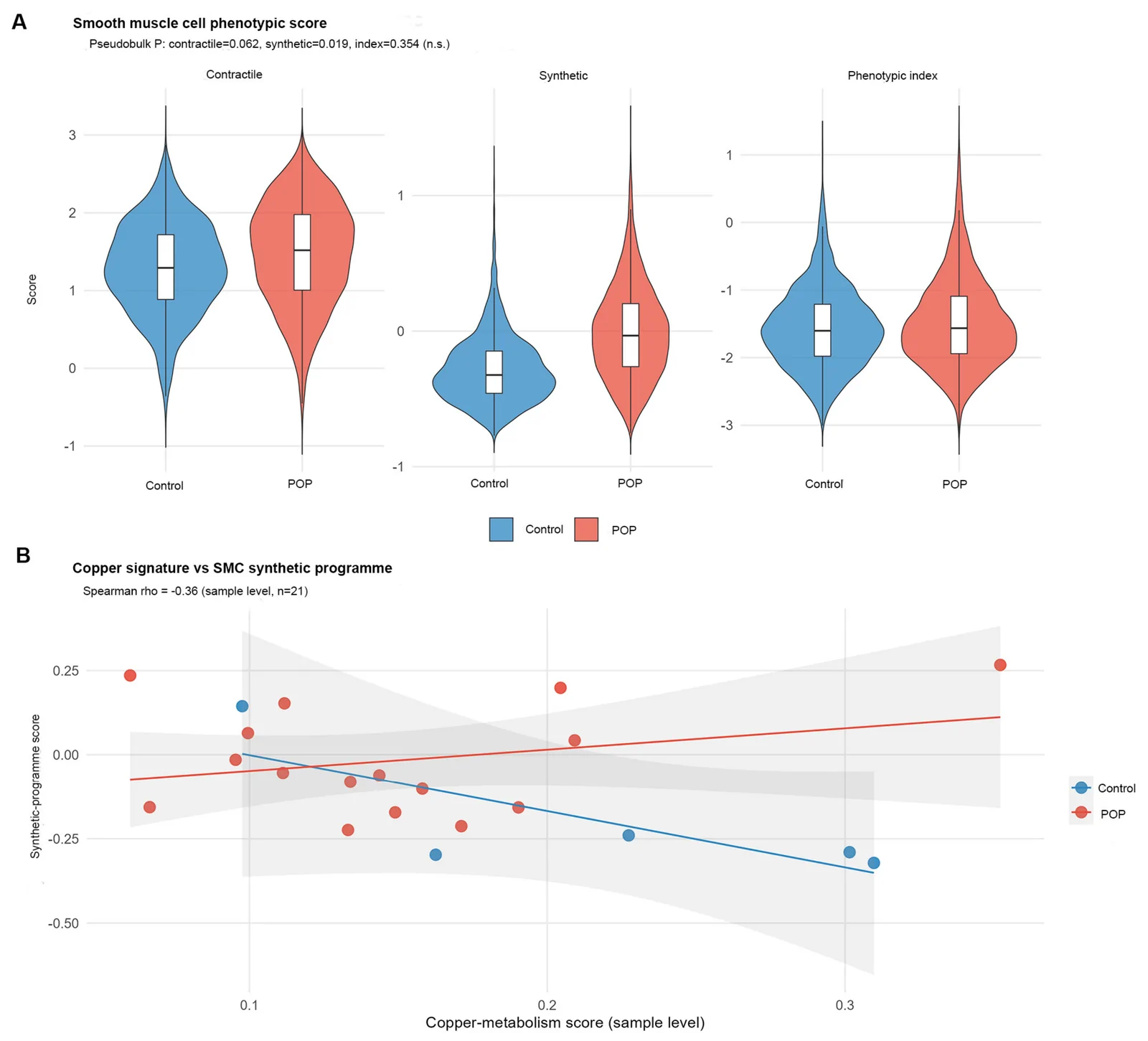
Smooth muscle cells phenotypic reprogramming. (**A**) Contractile, synthetic, and phenotypic index scores of control and POP smooth muscle cells (cell level and patient-level pseudobulk). (**B**) Correlation between patient-level copper metabolism scores and synthetic phenotype scores.

Using a single fixed gene set applied consistently across all analyses (Section 2.6), we found that POP SMCs shifted toward a synthetic phenotype. At the sample level (pseudobulk), the synthetic score was significantly elevated (control mean −0.201 vs. POP −0.017, *p* = 0.019), while the contractile score was elevated in the same direction (1.162 vs. 1.473, *p* = 0.062)—that is, both programs were upregulated rather than the contractile program remaining unchanged. The phenotypic index, defined as the synthetic score minus the contractile score, showed no significant difference at the sample level (−1.363 vs. −1.491, *p* = 0.354); we therefore do not use this index as evidence of phenotypic switching (Figure 4A, Table 5)

We then examined the relationship between the copper metabolism signature and SMCs phenotype. At the sample level, the copper metabolism score was negatively correlated with the synthetic score (rho ≈ −0.36 to −0.41) and weakly positively correlated with the phenotypic index (rho ≈ 0.17–0.20) (Figure 4B). In other words, within POP lesions, samples with higher copper signatures tended to have lower synthetic program scores, a relationship more consistent with a compensatory or counter-regulatory association.

### 3.7. Cell–Cell Communication

**Figure 5:**
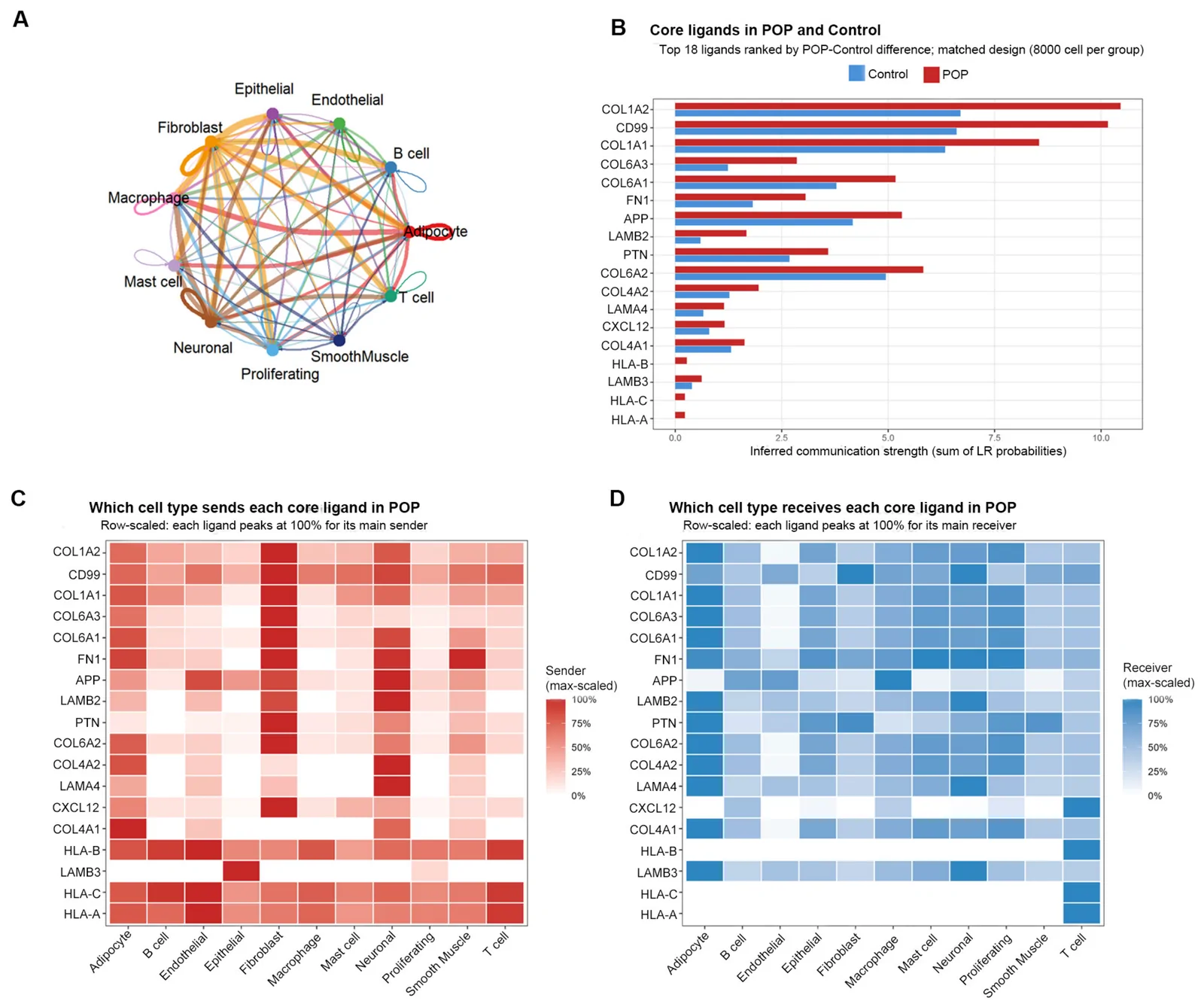
Cell–cell communication inferred by CellChat. (**A**) Circle plots of the overall inferred communication networks in the control and POP groups (CellChat). (**B**) Inferred communication strength of the top 18 ligands ranked by POP—control difference, shown as paired bars for the control and POP networks (strength = sum of probabilities of all ligand–receptor pairs carrying that ligand). (**C**) Sender atlas: for each core ligand, the cell types contributing most to its inferred signal in POP are shown (each row is scaled to the maximum value for that ligand, so the predominant sender for each ligand is 100%). (**D**) Receiver atlas, analogous to (**C**), showing the cell types receiving the most signal for each ligand.

CellChat analysis of the control and POP groups (8000 cells per group, matched by stratified downsampling) revealed reprogrammed cell–cell signaling in POP (Figure 5A). At the level of individual ligand–receptor pairs (Figure 5B), the strongest inferred signals in POP were carried by collagens and matrix glycoproteins: *COL1A2*, *CD99*, *COL1A1*, *COL6A1/6A2/6A3*, and *FN1* together accounted for the largest share of inferred communication strength, and each was higher than in controls (*COL1A2* Δ = +3.76, *CD99* Δ = +3.56, *COL1A1* Δ = +2.20, *COL6A3* Δ = +1.62, *COL6A1* Δ = +1.39, *FN1* Δ = +1.24). A second tier included *APP, LAMB2*, *PTN*, *COL4A1/4A2*, and *CXCL12*, all elevated in POP. A set of ligands was detected only in POP, including the matricellular protein *SPP1*, MHC class I ligands *HLA-A/B/C*, *COMP*, *TNXB*, and *TNF*; however, the absolute strength of these POP-only signals was an order of magnitude lower than that of the collagen ligands, so we report them as qualitative rather than quantitative findings. The complete ligand-level CellChat output is presented in Supplementary Table S15.

We separately resolved which cell types send each ligand (Figure 5C). Collagens *COL1A1/COL1A2* and *COL6A1/6A2/6A3*, together with *FN1, LAMB2*, and *APP*, were predominantly fibroblast derived, consistent with the ECM-centered interpretation of this study. *CD99* was mainly sent by immune populations (macrophages and mast cells), *SPP1* by macrophages, whereas *CXCL12* and MHC class I ligands originated mainly from the stromal and endothelial compartments. On the receiving side (Figure 5D), collagen ligands targeted fibroblasts, SMCs, and endothelial cells—that is, the principal ECM-producing and ECM-contacting compartments.

### 3.8. Trajectory Inference Within the Fibroblast Compartment

**Figure 6:**
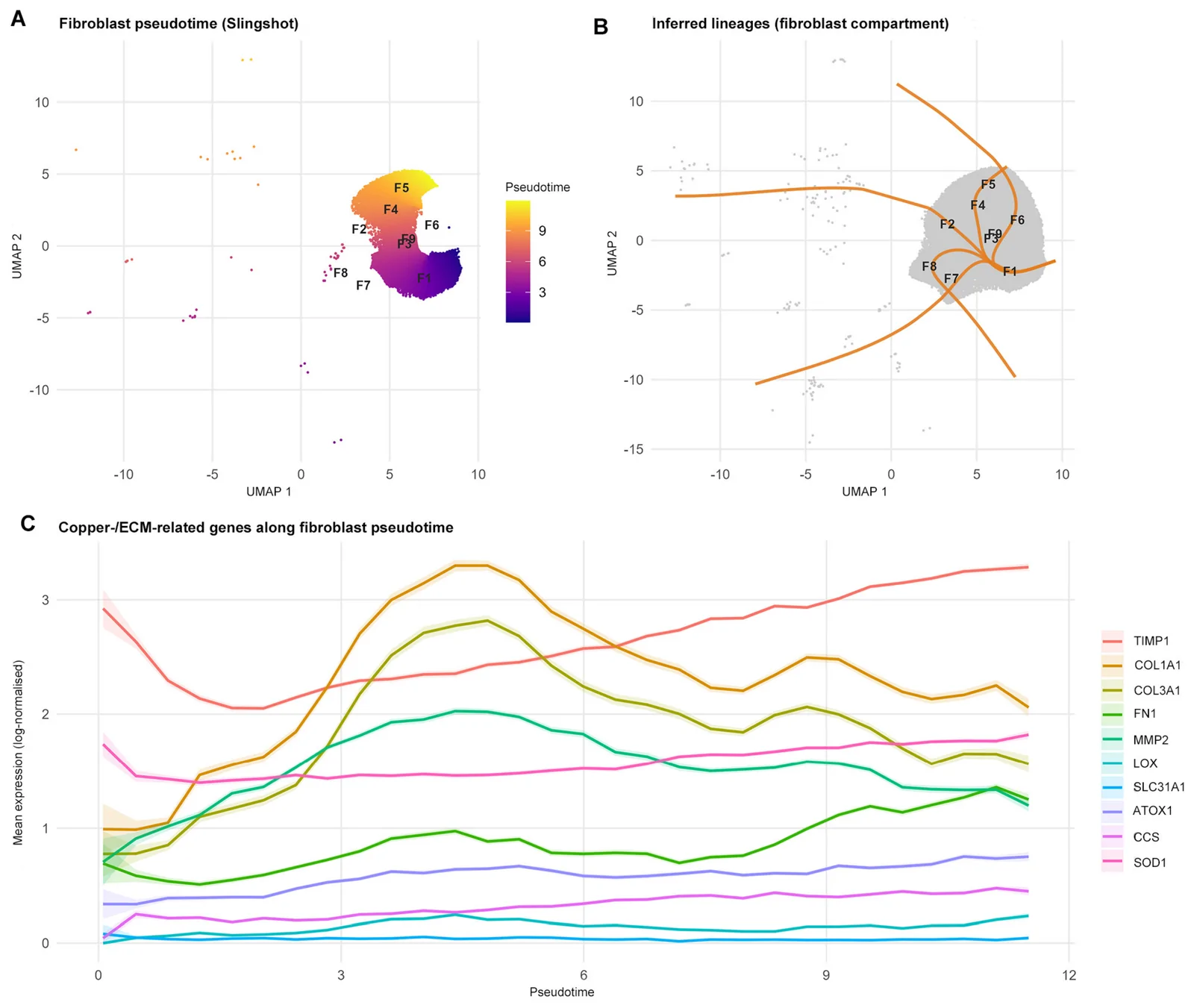
Trajectory inference within the fibroblast compartment. (**A**) Fibroblast UMAP colored by Slingshot pseudotime; the root state is the ECM-low subpopulation. (**B**) Inferred lineage curves on the same embedding. (**C**) Mean expression of copper-related and ECM-related genes along pseudotime.

Trajectory inference resolved several lineages and identified genes whose expression changes along inferred fibroblast states (Figure 6A,B). Among copper metabolism- and ECM-related genes (Figure 6C), *TIMP1* and *FN1* exhibited the most pronounced and sustained upward trends, reaching their highest levels at the pseudotime endpoint. *TIMP1* is a matrix metalloproteinase inhibitor and *FN1* is fibronectin. Their synchronous and sustained upregulation points to coordinated enhancement of ECM deposition and degradation inhibition—a core feature of the fibrotic phenotype. *COL1A1, COL3A1*, and *LOX* rose rapidly early in pseudotime, peaked at approximately pseudotime 3–4, and subsequently declined. This pattern suggests that ECM synthesis and crosslinking programs are activated early in the state transition but gradually attenuate at later stages. The *LOX* peak preceded the *COL1A1/COL3A1* peaks, consistent with the temporal logic of “crosslinking enzyme preceding substrate expression.” *SLC31A1* and *SOD1* showed little change early in pseudotime, began to rise after approximately pseudotime 6, and reached their highest levels at the endpoint. *SLC31A1* is a copper uptake channel and *SOD1* is a copper-dependent antioxidant enzyme. Their delayed upregulation suggests that activation of copper metabolism is a late event in the state transition, possibly representing a compensatory adaptation under sustained ECM secretion pressure.

The pseudotime origin (low ECM) can be regarded as a relatively quiescent fibroblast state, and the endpoint as a highly activated synthetic/fibrotic state. The dynamic changes along the trajectory suggest that the state transition of POP fibroblasts involves an ordered molecular program—early initiation of ECM synthesis and crosslinking (upregulation of *COL1A1*, *COL3A1*, *LOX*), mid-phase sustained enhancement of ECM deposition and degradation inhibition (sustained upregulation of *TIMP1, FN1*), and late activation of copper uptake and antioxidant defense (upregulation of *SLC31A1, SOD1*). However, the pseudotime curve is based on ordering inference from cross-sectional data; the “rise” or “fall” along the curve reflects expression differences among cells in different states, rather than true temporal changes within the same cell. In addition, the stable expression of *ATOX1* and *CCS* suggests that transcriptional regulation of copper chaperones may not be the principal regulatory layer of this pathway, and that protein stability or copper ion availability may instead be the rate-limiting factor.

### 3.9. Copper Homeostasis Imbalance Impairs Fibroblast Function

**Figure 7:**
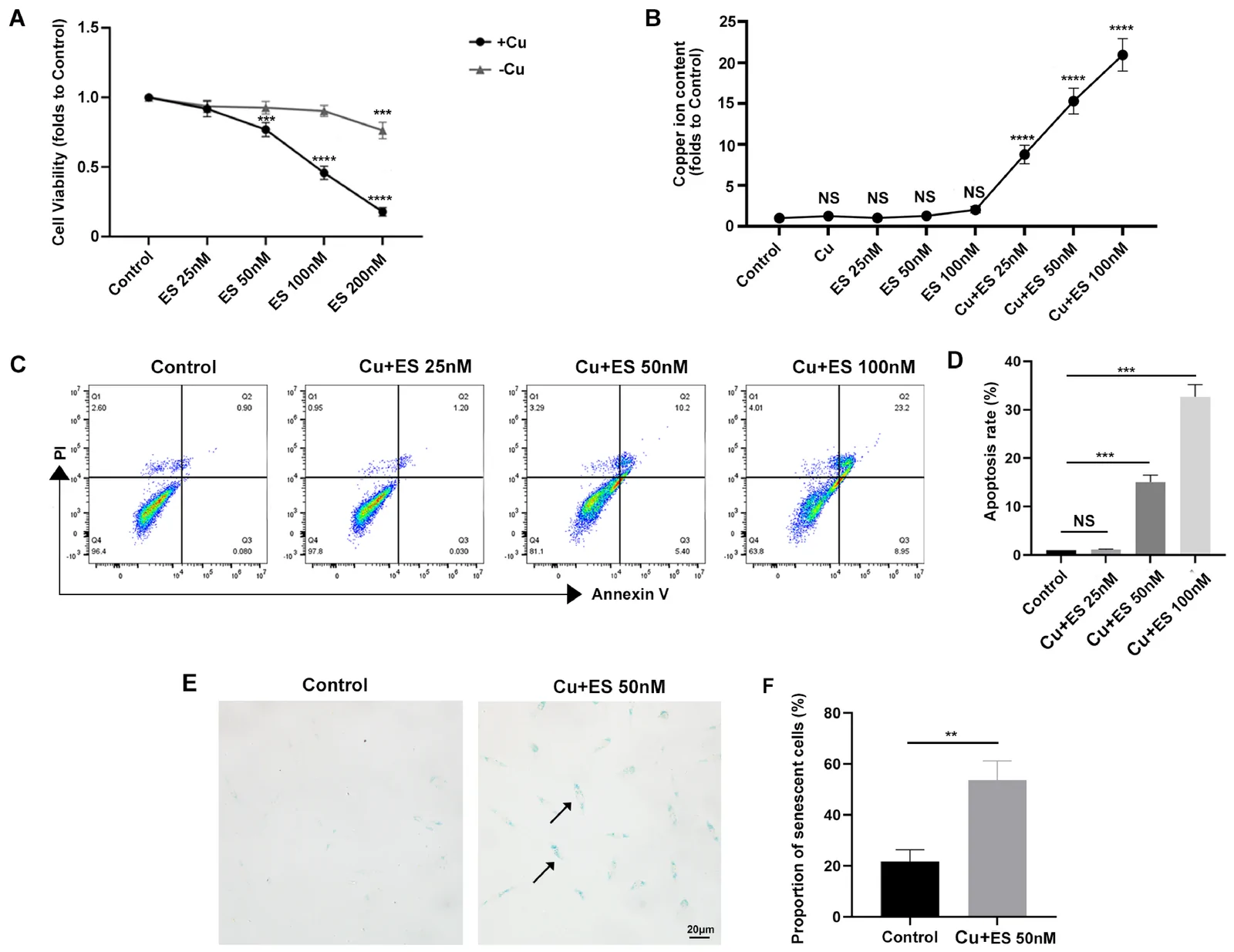
Copper homeostasis imbalance impairs L929 fibroblast function. (**A**) CCK-8 cell viability assay. L929 cells were treated with ES at 25, 50, 100, and 200 nM for 24 h under copper-supplemented (+Cu, filled circles) and copper-free (−Cu, open triangles) conditions. (**B**) Intracellular copper content measurement. Treatment conditions: control, Cu alone, ES alone (25, 50, 100 nM), and Cu+ ES (25, 50, 100 nM) co-treatment. (**C**) Apoptosis detection by Annexin V-FITC/PI flow cytometry. The color gradient from blue to red represents cell density (blue, low; red, high). Treatment conditions: control, Cu+ ES (25, 50, 100 nM) co-treatment. (**D**) Quantification of apoptotic rate; the sum of the upper-right and lower-right quadrants represents the apoptotic rate. (**E**) Representative images of SA-β-Gal staining. L929 cells were treated with vehicle control or Cu + ES 50 nM for 24 h and then subjected to SA-β-Gal staining; blue indicates senescence-positive cells (arrows indicate typical senescent cells; scale bar: 20 µm). (**F**) Quantification of the proportion of senescent cells. NS, no significance; ** *p* < 0.01; *** *p* < 0.001; **** *p* < 0.0001. ES, Elesclomol; Cu, 15 μM CuCl_2_.

Elesclomol reduced L929 fibroblast viability in a concentration- and copper-dependent manner (25–200 nM, 24 h; Figure 7A). Under co-treatment with 15 µM CuCl_2_, the inhibitory effect of ES on viability became significant from 50 nM (*p* < 0.001), and viability dropped to approximately 20% of control at 200 nM (*p* < 0.0001). In contrast, under copper-free conditions, the effect of ES on viability was markedly attenuated, with only a slight decrease at 200 nM (*p* < 0.001). This difference suggests that the cytotoxicity of ES depends largely on its mediated copper delivery, rather than on nonspecific toxicity of the ES molecule itself. Intracellular copper measurement directly validated the copper-delivery function of ES (Figure 7B): ES alone (25–100 nM) did not significantly increase intracellular copper, whereas Cu+ ES co-treatment produced significant ES concentration-dependent copper accumulation—intracellular copper was significantly elevated at 25 nM ES (*p* < 0.0001) and reached approximately 20-fold that of control at 100 nM (*p* < 0.0001). These results confirm that ES effectively promotes copper delivery into cells as a copper ionophore, and that this delivery is far stronger under Cu+ ES conditions than with Cu or ES alone, providing direct evidence for attributing the subsequent functional effects to copper dependence.

Apoptosis analyses were all performed with ES and copper co-treatment, confirming copper-dependent cell death. Annexin V/PI flow cytometry showed that the apoptotic rate at ES 25 nM did not differ significantly from control (NS), rose significantly to approximately 15% at ES 50 nM (*p* < 0.001), and further increased to approximately 33% at ES 100 nM (*p* < 0.001; Figure 7C,D). The concentration-dependent increase in apoptotic rate was consistent with the trend of declining viability, supporting apoptosis as the predominant form of ES-induced cell death. Senescence analysis showed that, in the presence of 15 µM CuCl_2_, SA-β-gal-positive cell proportion increased from approximately 20% in controls to approximately 55% after ES 50 nM treatment for 24 h (*p* < 0.01; Figure 7E,F). This result echoes the single-cell observation that the p53/senescence-like state F9 exhibited the largest decrease in copper metabolism score, suggesting that copper homeostasis imbalance not only induces acute cell death but also drives fibroblasts into a senescent state. Taken together across the three endpoints of viability, apoptosis, and senescence, this study demonstrates that copper homeostasis imbalance impairs fibroblast function in a copper-dependent manner, inducing apoptosis and senescence, and provides functional evidence for the involvement of copper metabolic dysregulation in stromal cell phenotypic remodeling in POP.

## 4. Discussion

The traditional pathological framework of POP has long centered on an imbalance between ECM synthesis and degradation. The data in this study offer an important revision to this framework: although ECM remodeling was robustly elevated in POP fibroblasts across four independent scoring methods (4/4), copper metabolism scores were robustly decreased (3/4), and copper metabolism activity was completely decoupled from ECM remodeling scores at the patient level (rho = −0.02, *p* = 0.92). This “decoupling” phenomenon is one of the most critical findings of this study—it suggests that copper metabolic dysregulation may constitute a regulatory dimension in POP stromal pathology that is independent of the classical ECM axis.

This finding forms a thought-provoking contrast with the established copper–LOX research in the POP field. LOX, a copper-dependent monoamine oxidase, is a key enzyme for the covalent crosslinking of collagen and elastic fibers [17,18]. In the classical model of POP, insufficient LOX function leads to impaired crosslinking of pelvic floor connective tissue, thereby weakening the mechanical strength of supporting structures [19,20]. However, the trajectory analysis in this study reveals a more complex picture: *LOX* expression peaked early during the fibroblast state transition, whereas upregulation of the copper uptake channel *SLC31A1* and the copper-dependent antioxidant enzyme *SOD1* was delayed until late pseudotime. This implies that, in POP fibroblasts, transcriptional activation of *LOX* may precede the restoration of copper supply capacity—a temporal mismatch of “enzyme in place, cofactor delayed.” This temporal pattern offers a new mechanistic hypothesis for how copper metabolic dysregulation participates in POP: the problem may not lie in insufficient *LOX* expression, but rather in the inability of the copper delivery system to provide sufficient copper cofactor to *LOX* within the required time window.

The present study found that fibroblast states with high copper metabolism activity were not enriched for ECM programs, but were instead dominated by TNFA_SIGNALING_VIA_NFKB, accompanied by enrichment of hypoxic, EMT, and oxidative phosphorylation programs. At the patient level, copper metabolism activity was positively correlated with senescence scores (rho = 0.56, *p* = 0.010), and this association was stronger within the POP stratum (rho = 0.65) and completely absent within the control stratum (rho = 0.00). This finding liberates copper from its role as a “supplier of cofactors for ECM crosslinking” and assigns it a new identity as a regulatory node of the inflammation–senescence axis. This association has a plausible mechanistic basis in aging biology. Copper is an essential cofactor for mitochondrial oxidative phosphorylation, and mitochondrial dysfunction is one of the core drivers of cellular senescence [21,22,23]. Senescent fibroblasts secrete factors such as IL-6 and TNF-α through the senescence-associated secretory phenotype (SASP), forming autocrine and paracrine inflammatory loops [24,25]. Macrophage-derived inflammatory signals can likewise induce senescence-associated transcriptional programs in neighboring fibroblasts, including the expression of NF-κB-regulated long noncoding RNAs [26]. In the data of this study, high-copper-metabolic-activity fibroblasts were simultaneously enriched for TNFα/NF-κB signaling and oxidative phosphorylation programs, suggesting that copper metabolic activity may reflect an intersection between mitochondrial functional status and inflammatory signaling—cells with adequate copper supply maintain higher oxidative metabolic activity, and this activity is redirected toward NF-κB-dependent inflammation–senescence programs in the inflammatory microenvironment of POP.

It is worth noting that the positive correlation between copper metabolism activity and senescence scores in POP appears to contradict the classical expectation that copper deficiency leads to LOX inactivation in ECM remodeling. This apparent “contradiction” precisely reveals the cell-type specificity and functional duality of copper in POP: at the level of ECM crosslinking, insufficient copper may impair LOX function; at the level of cell state regulation, higher copper metabolic activity is coupled to inflammation–senescence programs. The two are not facets of the same biological process, but rather manifestations of copper acting in different subcellular and functional modules. In vitro experiments provided causal evidence for the above associations: the copper ionophore ES reduced fibroblast viability and increased the proportion of apoptotic and senescent cells in a copper-dependent manner. ES, as a well-studied copper ionophore, can induce copper-dependent cell death (cuproptosis) by delivering copper to mitochondria, a mechanism involving FDX1-dependent oligomerization of lipoylated proteins [27]. However, the effects observed in this study—decreased viability accompanied by apoptosis and senescence—suggest that ES within this concentration range (25–100 nM) may not fully activate the classical cuproptosis pathway, but instead induces a stress response dominated by senescence. This forms an intriguing echo with the single-cell observation that high-copper-metabolic-activity fibroblasts are enriched for the senescence-like state F9 (which showed the largest decrease in copper score, Δ = −0.078): whether “high copper” or “copper dysregulation,” both ultimately converge on a senescent phenotype.

The SMCs data extend copper metabolic dysregulation from fibroblasts to the entire stromal cell compartment. In POP, SMCs shifted toward a synthetic phenotype (*p* = 0.019), and SMC copper metabolism scores were negatively correlated with synthetic scores (rho ≈ −0.36 to −0.41). This contrasts with the pattern of “copper metabolism decoupled from ECM scores” observed in fibroblasts: in SMCs, the negative correlation between copper metabolism and the synthetic phenotype suggests that copper dysregulation may be more directly involved in SMCs phenotypic switching. Atox1-mediated copper delivery has been shown to regulate LOX activity and cell migration in vascular SMCs [28], and its deficiency leads to suppressed neointima formation and reduced ECM expansion. The co-occurrence of decreased SMCs copper metabolism and synthetic shift in POP may reflect a process of copper demand redistribution during the transition from a contractile to a synthetic phenotype—the proliferative and migratory demands of synthetic SMCs may alter the allocation priority of copper between LOX activation and cellular metabolism.

Several limitations of this study should be acknowledged. First, the functional validation in this study was based on the L929 mouse fibroblast cell line, which differs from primary human pelvic floor fibroblasts in species and tissue origin; therefore, the copper-dependent effects of Elesclomol should be regarded as proof-of-principle validation rather than a quantitative prediction of the efficacy of copper-targeted intervention in pelvic floor fibroblasts. Moreover, all functional experiments were performed in vitro and cannot fully recapitulate the complex mechanical environment and intercellular communication network of POP tissue [29]; the present study did not evaluate the regulatory effect of mechanical stimulation on copper metabolism. Second, changes in cell proportions did not reach FDR-corrected significance at the patient level, so conclusions regarding subpopulation compositional changes should be regarded as trend-level findings. Third, single-cell copper metabolism scores are based on transcript levels and cannot reflect the actual subcellular distribution of copper ions or the functional status of copper proteins—the stable expression of *ATOX1* and *CCS* suggests that transcriptional regulation of copper chaperones may not be the rate-limiting step, and that protein stability or copper ion availability may instead be critical. Future studies should aim to measure the copper pool and its subcellular distribution in POP fibroblasts, evaluate the regulation of copper transporters by mechanical stretch, and validate the interventional effects of copper chelators or ionophores on the mechanical properties of pelvic floor supporting tissues in animal models, thereby further elucidating the detailed regulatory mechanisms of copper metabolism in POP pathogenesis.

## 5. Conclusions

This study, through integrated single-cell transcriptomic analysis, reveals that stromal cells in the anterior vaginal wall of POP undergo phenotypic remodeling characterized by enhanced ECM remodeling and decreased copper metabolism, elevating copper metabolism from a long-neglected “cofactor supply” layer in POP pathology to an independent dimension of stromal cell phenotypic regulation. The core finding that copper metabolism is decoupled from ECM programs and coupled to inflammation–senescence programs challenges the unidirectional linear model in which copper dysregulation leads to LOX inactivation and subsequent ECM crosslinking impairment, suggesting that copper homeostasis imbalance in POP may contribute to stromal microenvironment remodeling through activation of the inflammation–senescence axis, rather than (or not solely through) impaired ECM crosslinking. This framework provides a new path for understanding the molecular pathology of POP and offers preliminary functional evidence for the potential value of copper metabolism-targeted intervention strategies.

## Figures and Tables

**Table 1:** Gene set lists.

Gene Set	Gene Count	Representative Genes
Copper metabolism	21	*SLC31A1*, *ATP7A*, *ATP7B*, *ATOX1*, *CCS*, *COX17*, *LOX*, *SOD1*, *MT1A*, *MT2A*
ECM remodeling	20	*COL1A1*, *COL3A1*, *FN1*, *MMP2*, *TIMP1*, *LOX*
Cellular senescence	17	*CDKN1A*, *CDKN2A*, *TP53*, *IL6*, *TGFB1*
Mitochondrial damage	31	*MT-ND1-6*, *MFN1*, *MFN2*, *SOD2*
Cuproptosis	9	*FDX1*, *LIAS*, *DLAT*, *DLST*

**Table 2:** Cell count and proportion of each cell type in Control and POP groups.

Cell Type	Control (n = 17,416)	POP (n = 73,162)	*p* (Sample Level)
Fibroblast	7334 (42.1%)	40,103 (54.8%)	0.153
Smooth muscle cell	3170 (18.2%)	7560 (10.3%)	0.313
Adipocyte	1544 (8.9%)	6269 (8.6%)	0.603
Macrophage	1020 (5.9%)	5937 (8.1%)	0.240
Endothelial cell	2546 (14.6%)	5740 (7.8%)	0.050
T cell	728 (4.2%)	3247 (4.4%)	0.398
B cell	588 (3.4%)	1665 (2.3%)	1.000
Epithelial cell	93 (0.5%)	1383 (1.9%)	0.123
Proliferating cell	136 (0.8%)	829 (1.1%)	0.091
Neuronal cell	39 (0.2%)	252 (0.3%)	0.603
Mast cell	218 (1.3%)	177 (0.2%)	0.206

**Table 3:** Module scores in fibroblasts.

Module	AddModuleScore (*p*)	AUCell (*p*)	Pseudobulk-z (*p*)	Depth-Matched (*p*)	Direction
Copper metabolism	Decreased (≈0)	Decreased (≈0)	Increased (0.40)	Decreased (1 × 10^−5^)	Decreased (robust)
ECM remodeling	Increased (1.7 × 10^−168^)	Increased (4.4 × 10^−253^)	Increased (0.18)	Increased (0.19)	Increased (robust)
Cellular senescence	Decreased (≈0)	Decreased (≈0)	Decreased (0.35)	Decreased (1 × 10^−5^)	Decreased (robust)
Mitochondrial damage	Increased (2.2 × 10^−37^)	Decreased (0.28)	Increased (0.15)	Increased (0.009)	Inconsistent
Cuproptosis	Increased (0.053)	Decreased (0.72)	Decreased (0.90)	Increased (0.19)	Inconsistent

**Table 4:** Fibroblast subpopulations after re-clustering.

State	Cell Number	Representative Marker Genes	Annotation
F1	8330	*WNT16*, *CSF3*, *LIF*, *HMOX1*, *CXCL1*, *GCLM*, *TXNRD1*	Inflammatory/stress (NF-κB + NRF2)
F2	7010	*COCH*, *COMP*, *GREM1*, *FIBIN*, *LTBP1*, *HOXA10*	Matrix/chondroid-like
F3	6362	*SOCS3*, *DNAJB1*, *IRF1*, *JUNB*, *FOSB*, *ATF3*, *HSPA1B*	Immediate early gene/heat shock
F4	5924	*C7*, *ADH1B*, *RSPO3*, *AGTR1*, *MGP*, *FRZB*	Complement/vascular
F5	5551	*CLEC3B*, *MFAP5*, *VIT*, *SCARA5*, *PI16*, *CD34*	Quiescent/progenitor
F6	5045	*HAS1*, *CLEC3B*, *ACKR3*, *VIT*, *SCARA5*, *MEDAG*	Progenitor, HAS1+
F7	4138	*POSTN*, *ADAM12*, *MMP11*, *RUNX2*, *WNT5A*, *LOXL2*, *TNC*	Myofibroblast/pro-fibrotic
F8	3582	*NKD2*, *APCDD1*, *STRA6*, *APOD*, *LEPR*, *HOPX*	Adipogenic precursor
F9	1495	*RARRES1*, *DKK1*, *FDXR*, *PHLDA3*, *ZMAT3*, *DDB2*	p53/senescence-like

**Table 5:** Smooth muscle cells phenotypic scores.

Score	Cell-Level Median (Control vs. POP)	Pseudobulk Mean (Control vs. POP)	Pseudobulk *p*
Contractile	1.291 vs. 1.517	1.162 vs. 1.473	0.062
Synthetic	−0.324 vs. −0.033	−0.201 vs. −0.017	0.019
Phenotypic index	−1.603 vs. −1.565	−1.363 vs. −1.491	0.354

## Data Availability

The single-cell dataset analyzed in this study (GSE151192) is publicly available from GEO (https://www.ncbi.nlm.nih.gov/geo, accessed on 30 July 2026). The analytical codes used in this study are available from the corresponding author upon reasonable request.

## Supplementary Materials

The following supporting information can be downloaded at: https://www.mdpi.com/article/10.3390/biology15201841/s1, Table S1. Copper-metabolism gene set (21 genes) with the functional annotation of each member. Table S2. ECM-remodeling gene set (20 genes) with per-gene functional annotation. Table S3. Cell-senescence gene set (18 genes) with per-gene functional annotation. Table S4. Mitochondrial-damage gene set (32 genes) with per-gene functional annotation. Table S5. Cuproptosis gene set (9 genes) with per-gene functional annotation and reference presence/absence. Table S6. Summary of the five functional gene sets. For each set: the number of curated genes. Table S7. Complete marker genes of the nine fibroblast subpopulations (F1–F9). Table S8. Fibroblast subpopulation identities and annotation. Table S9. Fibroblast subpopulation abundance. Table S10. Copper-metabolism score by fibroblast subpopulation (F1–F9). Table S11. Differentially expressed genes between high- and low-copper fibroblasts (within-patient median split). Table S12. Fgsea enrichment of Hallmark gene sets between high- and low-copper fibroblasts. Table S13. Fgsea enrichment of GO Biological Process gene sets between high- and low-copper fibroblasts. Table S14. Cross-module correlation matrix (AUCell). Table S15. Ligand-level CellChat output.

## Abbreviations

The following abbreviations are used in this manuscript:

POP	Pelvic organ prolapse
DALY	Disability-adjusted life year
ECM	Extracellular matrix
SMCs	Smooth muscle cells
LOX	Lysyl oxidase
GEO	Gene Expression Omnibus
UMAP	Uniform Manifold Approximation and Projection
DEGs	Differentially expressed genes
ES	Elesclomol
